# Health-related quality of life and disease activity in rheumatoid arthritis: a cross-sectional study in West Java, Indonesia

**DOI:** 10.3389/fmed.2025.1682924

**Published:** 2025-10-30

**Authors:** Yudisia Ausi, Rano K. Sinuraya, Sumartini Dewi, Melisa I. Barliana, Maarten J. Postma, Auliya A. Suwantika

**Affiliations:** ^1^Doctoral Program of Pharmacy, Faculty of Pharmacy, Universitas Padjadjaran, Jatinangor, Indonesia; ^2^Department of Biological Pharmacy, Faculty of Pharmacy, Universitas Padjadjaran, Jatinangor, Indonesia; ^3^Department of Pharmacology and Clinical Pharmacy, Faculty of Pharmacy, Universitas Padjadjaran, Jatinangor, Indonesia; ^4^Center of Excellence for Pharmaceutical Care Innovation, Universitas Padjadjaran, Bandung, Indonesia; ^5^Division of Rheumatology, Department of Internal Medicine, Faculty of Medicine, Universitas Padjadjaran, Jatinangor, Indonesia; ^6^Hasan Sadikin General Hospital, Bandung, Indonesia; ^7^Unit of Global Health, Department of Health Sciences, University Medical Center Groningen, University of Groningen, Groningen, Netherlands; ^8^Center for Health Technology Assessment, Universitas Padjadjaran, Bandung, Indonesia

**Keywords:** RA, EQ-5D, DAS28, QoL, disease activity

## Abstract

**Background:**

Joint inflammation is a key manifestation of rheumatoid arthritis (RA), often leading to functional limitations and reduced quality of life (QoL) as the disease progresses. This study aims to examine the association between disease activity and health-related quality of life (HRQoL) in RA patients.

**Methods:**

A cross-sectional study was conducted among 110 RA patients aged 18–65, diagnosed according to the 2010 ACR/EULAR criteria and receiving disease-modifying antirheumatic drugs (DMARDs) therapy for at least 1 month. Patient data were obtained from electronic medical records and verified through structured interviews. Health-related quality of life (HRQoL) was assessed using the EQ-5D-5L questionnaire, while disease activity was measured using the DAS28-ESR. Non-parametric analysis was performed to compare EQ-5D scores based on patient characteristics.

**Result:**

The most frequently reported problem was pain/discomfort (76.36%), showing significant differences across disease activity groups. Other commonly affected dimensions included usual activities (46.36%), anxiety/depression (44.55%), mobility (33.64%), and self-care (19.09%). The mean EQ-5D index was 0.76 (95% CI, 0.73–0.80), and the visual analogue scale (VAS) score was 73.05 (95% CI, 69.91–76.19). Disease activity and comorbidity showed a statistically significant association with HRQoL (*p* = <0.001; OR = 6.33; CI 95% 2.29–17.51 and *p* = 0.005; OR = 3.65; 95% CI 1.50–8.93, respectively).

**Conclusion:**

This study suggests that higher disease activity and comorbidity were negatively associated with HRQoL among RA patients. Disease progression disrupts multiple dimensions of quality of life of the subjects. This study highlights the importance HRQoL assessments in RA management to ensure a more comprehensive approach to patient care.

## 1 Introduction

Rheumatoid arthritis (RA) is a chronic autoimmune disease characterized by symmetrical inflammatory polyarthritis, initially affecting the small joints of the hands and feet, and progressively involving larger joints as the disease advances (1). Its etiology is multifactorial, involving both genetic predisposition and environmental triggers (2). Common clinical manifestations include morning stiffness, joint pain, intra-articular effusion, periarticular swelling, reduced joint mobility, and muscle weakness (3). Without appropriate treatment, RA can lead to chronic pain, irreversible joint deformities, and substantial functional impairment (4).

Beyond physical symptoms, RA exerts a profound impact on patients’ psychological well-being and social functioning. The long-term nature of the disease, combined with physical limitations, often disrupts coping mechanisms and may result in depression, ultimately worsening disease outcomes (5). Consequently, RA significantly impairs health-related quality of life (QoL), a multidimensional construct encompassing physical, psychological, and social well-being (6).

Timely diagnosis and early initiation of disease-modifying antirheumatic drugs (DMARDs) are essential strategies for controlling disease activity and preventing joint destruction. Among conventional synthetic DMARDs (csDMARDs), methotrexate (MTX) is the most widely prescribed, used in approximately 69.9% of cases (7). These medications help suppress inflammation and reduce disease activity, thereby alleviating pain and limiting joint damage.

Despite the growing emphasis on patient-centered care, few quantitative studies in Indonesia have explored RA patients’ perspectives on HRQoL (8). HRQoL is often assessed using patient-reported outcome measures to evaluate the impact of chronic illness and the effectiveness of therapeutic interventions (9). Understanding patients’ lived experiences is essential to optimizing care, especially in the context of chronic, disabling conditions such as RA. This study aims to examine the association between disease activity and HRQoL among RA patients in Indonesia, focusing on Bandung—one of the country’s most populous urban centers—as a reference setting.

## 2 Methods

### 2.1 Study setting and population

This study was conducted at Hasan Sadikin General Hospital, the primary public referral center in Bandung, West Java, Indonesia. A total of 110 patients with rheumatoid arthritis (RA) were recruited between October and December 2023. This sample size complied with the minimum requirement, which was pre-determined using single proportion formula at a 95% confidence level. Eligible participants met the following criteria: (i) aged 18–65 years; (ii) diagnosed with RA according to the 2010 classification criteria from the American College of Rheumatology/European League Against Rheumatism (ACR/EULAR); and (iii) had been receiving disease-modifying antirheumatic drugs (DMARDs) for at least 1 month. Patients with chronic infections (e.g., HIV, hepatitis B, or tuberculosis) or incomplete medical records were excluded. Written informed consent was obtained from all participants prior to enrollment.

### 2.2 Data collection and analysis

Patient data were obtained from medical records and verified through interviews. Health-related quality of life (HRQoL) was assessed using the EQ-5D-5L questionnaire, while disease activity was measured using the Disease Activity Score in 28 joints with erythrocyte sedimentation rate (DAS28-ESR).

The EQ-5D-5L is a brief, generic self-assessment tool for HRQoL. It consists of five items, each rated on a five-level Likert scale, where level 1 indicates no problems and level 5 indicates extreme problems. The five domains assessed are mobility, self-care, usual activities, pain/discomfort, and anxiety/depression. In addition to the five-domain profile, the instrument includes a visual analogue scale (VAS), which allows patients to rate their overall health status on a scale from 0 (worst imaginable health) to 100 (best imaginable health). Participants completed the paper-based version of the questionnaire, which had been translated into Bahasa Indonesia (10). The EQ-5D outputs include a utility score (ranging from 0 to 1) and a VAS score (ranging from 0 to 100). The utility score was calculated using the Indonesian value set, where 0 represents death and 1 represents perfect health (10).

DAS28-ESR is a composite index used to assess RA disease activity. It incorporates the number of tender and swollen joints (out of 28), VAS of limited motion, and the erythrocyte sedimentation rate (ESR). Disease activity is categorized as follows: remission (DAS28 < 2.6), low disease activity (2.6 ≤ DAS28 ≤ 3.1), moderate disease activity (3.1 < DAS28 < 5.1), and high disease activity (DAS28 ≥ 5.1) (11). The difference of EQ-5D scores across patient characteristics and disease activity groups was analyzed using the Mann–Whitney U test for dichotomous variables and the Kruskal–Wallis test for polytomous variables. Differences in the proportions of reported problems across EQ-5D domains by disease activity groups were examined using the chi-square test. Multivariate analysis and odds ratio calculation were conducted using binary logistic regression with median of EQ-5D as the cut-off value to categorize the outcome. A *p*-value less than 0.05 indicates statistical significance. Variables with *p* < 0.25 in bivariate analysis were included in multivariate model. Statistical analysis was performed using IBM SPSS v.27.

## 3 Results

Among the included subjects, the majority were women (*n* = 102; 92.7%), with a mean age of 43.86 years (SD = 11.48). All participants were Indonesian, and 81 (73.6%) identified as Sundanese, the indigenous ethnic group native to West Java. Regarding lifestyle factors, only 6.4% were active smokers, and none reported current alcohol consumption. Almost half of patients (*n* = 54, 48.6%) had chronic disease comorbidity (Table 1).

**Table 1:** Sociodemographic and clinical characteristics of patients.

Variable	Value
Sex (*n*, %)	
Female	102 (92.7%)
Male	8 (7.3%)
Age, year (Mean, SD)	43.86 ± 11.48
Age (*n*, %)	
< 44 years	78 (70.9%)
≥ 44 years	32 (29.1%)
Ethnicity (*n*, %)	
Sundanese	81 (73.6%)
Non-Sundanese	29 (26.4%)
Smoking history (*n*, %)	
Non-smoker	94 (85.5%)
Former smoker	9 (8.2%)
Smoker	7 (6.4%)
Alcoholic history (*n*, %)	
Non-alcoholic	107 (97.3%)
Former alcoholic	3 (2.7%)
Age of onset (Mean, SD)	38.58 ± 11.86
Age of onset (*n*, %)	
<30	22 (20.0%)
30–55	81 (73.6%)
>55	7 (6.4%)
Chronic comorbidities (*n*, %)	
No	56 (50.5%)
Yes	54 (48.6%)
Disease duration, year (Median, IQR)	3 (6.25)
Disease duration (*n*, %)	
<3 years	51 (46.4%)
≥3 years	59 (53.6%)
Current therapy (*n*, %)	
MTX monotherapy	66 (60.0%)
non-MTX monotherapy	7 (6.4%)
2 csDMARDs	32 (29.1%)
3 csDMARDs	5 (4.5%)
Type of DMARDs in use (*n*, %)	
Methotrexate	96 (87.3%)
Azathioprine	4 (3.6%)
Hydroxychloroquine or Chloroquine	23 (20.9%)
Sulfasalazine	14 (12.7%)
Leflunomide	11 (10.0%)
Cyclosporine	4 (3.6%)
Weekly MTX dose, tablets weekly ^a^ (Median, IQR)	5 (4)
Methyl Prednisolone dose, mg daily (Median, IQR)	4 (2)
Methyl prednisolone dose (*n*, %)	
< 4 mg daily	37 (33.6%)
≥4 mg daily	73 (66.4%)
Involuntary discontinuation of MTX (*n*, %)	
No involuntary discontinuation history	83 (75.5%)
Discontinuation history due to intolerance	13 (11.8%)
Discontinuation history due to lack of effectiveness	3 (2.7%)
Discontinuation history due to other reasons	11 (10.0%)
Rheumatoid factor (*n*, %)	
Reactive	47 (42.7%)
Non-reactive	62 (56.4%)
Unknown	1 (0.9%)
Duration of MTX therapy (*n*, %)	
< 6 months	26 (23.6%)
6–12 months	26 (23.6%)
>12 months	58 (52.7%)
DAS28-ESR (Mean, SD)	2.87 ± 0.99
Disease activity category (*n*, %)	
Remission	49 (44.55%)
Low disease activity	27 (24.55%)
Moderate disease activity	32 (29.09%)
High disease activity	2 (1.82%)

^a^ MTX tablet 2.5 mg.

SD, standard deviation; IQR, interquartile range; MTX, methotrexate; DMARDs, disease-modifying antirheumatic drugs; csDMARDs, conventional synthetic disease-modifying antirheumatic drugs; DAS28, Disease activity score 28; ESR, Erythrocyte sedimentation rate.

Most patients were diagnosed with rheumatoid arthritis (RA) between the ages of 30 and 55 (*n* = 81; 73.6%), a range classified as young-onset rheumatoid arthritis (YORA) (12). Median disease duration was 3 years (IQR = 6.25). In terms of pharmacological treatment, methotrexate (MTX) monotherapy was the most common regimen (*n* = 66; 60.0%), with a median dose of five tablets per week (equivalent to 12.5 mg) (IQR = 4). Over half of the patients (*n* = 58; 52.7%) had been using MTX for more than 1 year. Most patients used MTX in their current treatment (*n* = 96; 87.3%). Other types of csDMARDs were also used, such as Azathioprine (3.6%), Hydroxychloroquine or Chloroquine (20.9%), Sulfasalazine (12.7%), Leflunomide (10.0%), and Cyclosporine (3.6%). No subject received biological or targeted synthetic DMARDs as routine therapy for RA patients. Methylprednisolone was prescribed at a median dose of 4 mg/day (IQR = 2). All patients also received folic acid and calcium supplementation. Regarding rheumatoid factor (RF) status, more than half of the patients were RF negative in the beginning of treatment (*n* = 62; 56.4%). Additional details on the sociodemographic and clinical characteristics of the respondents are provided in Table 1.

In terms of disease activity, most patients achieved remission (*n* = 49; 44.6%), while 27 (24.6%) had low disease activity (LDA). In contrast, 32 patients (29.1%) had moderate disease activity (MDA), and two (1.8%) remained in a state of high disease activity (HDA) (Table 1).

When examining health-related quality of life (HRQoL) domains, mild to severe problems in self-care were more frequently reported among patients with higher disease activity (MDA and HDA) compared to those with lower activity (LDA and remission) (*p* = 0.03). A similar pattern was observed in the mobility domain (*p* < 0.01), although no severe problems were reported in this domain (Table 2).

**Table 2:** Cross tabulation of EQ-5D score and disease activity groups.

Domain	No problem		Mild		Moderate		Severe		Unable		*p-*value
	*n*	%	*n*	%	*n*	%	*n*	%	*n*	%	
Mobility											
Lower disease activity group	60	54.55%	15	13.64%	1	0.91%	0	0.00%	0	0.00%	<0.01*
Higher disease activity group	13	11.82%	16	14.55%	5	4.55%	0	0.00%	0	0.00%	
Total	**73**	66.36%	**31**	28.18%	**6**	5.45%	**0**	0.00%	**0**	0.00%	
Self-care											
Lower disease activity group	68	61.82%	8	7.27%	0	0.00%	0	0.00%	0	0.00%	0.03*
Higher disease activity group	21	19.09%	10	9.09%	2	1.82%	1	0.91%	0	0.00%	
Total	**89**	80.91%	**18**	16.36%	**2**	1.82%	**1**	0.91%	**0**	0.00%	
Usual activities											
Lower disease activity group	50	45.45%	22	20.00%	4	3.64%	0	0.00%	0	0.00%	<0.01*
Higher disease activity group	9	8.18%	17	15.45%	8	7.27%	0	0.00%	0	0.00%	
Total	**59**	53.64%	**39**	35.45%	**12**	10.91%	**0**	0.00%	**0**	0.00%	
Pain/Discomfort											
Lower disease activity group	26	23.64%	38	34.55%	10	9.09%	2	1.82%	0	0.00%	<0.01*
Higher disease activity group	0	0.00%	12	10.91%	20	18.18%	2	1.82%	0	0.00%	
Total	**26**	23.64%	**50**	45.45%	**30**	27.27%	**4**	3.64%	**0**	0.00%	
Anxiety/Depression											
Lower disease activity group	46	41.82%	16	14.55%	10	9.09%	4	3.64%	0	0.00%	0.391
Higher disease activity group	15	13.64%	9	8.18%	8	7.27%	2	1.82%	0	0.00%	
Total	**61**	55.45%	**25**	22.73%	**18**	16.36%	**6**	5.45%	**0**	0.00%	

Lower disease activity group = patients with remission and low disease activity (LDA); Higher disease activity group = patients with moderate disease activity (MDA) and high disease activity (HDA); * Chi-square test, *p* < 0.05 considered statistically significant; bold values indicate statistically significant results.

Across all respondents, the most frequently reported problems (mild to severe) were in the pain/discomfort domain (76.4%), followed by usual activities (46.4%), anxiety/depression (44.6%), mobility (33.6%), and self-care (19.1%). No participant reported a level 5 (extreme/unable) problem in any domain. However, level 4 (severe) problems were noted, particularly in anxiety/depression (5.5%), pain/discomfort (3.6%), and self-care (0.9%) (Table 2).

The overall mean EQ-5D index score was 0.76 (95% CI, 0.73–0.80), with a comparable mean EQ-5D VAS score of 73.05 (95% CI, 69.91–76.19) (Table 3). EQ-5D index scores varied significantly by disease activity group. Patients with higher disease activity (MDA and HDA) reported significantly lower EQ-5D scores compared to those in remission or with low disease activity (*p* < 0.001, Table 4). These findings confirm a significant association between disease activity and HRQoL in Indonesian population. Bivariate analysis also showed a significant association between comorbidities and HRQoL (*p* = 0.001). No significant association was observed between HRQoL and other sociodemographic or clinical characteristics (Table 4).

**Table 3:** Quality of life among overall patients (*n* = 110).

Variable	Mean	SD	Median	IQR
EQ-5D score	0.76	0.18	0.79	0.27
EQ-5D VAS	73.05	16.62	80.00	21

SD, standard deviation; IQR, interquartile range; VAS, visual analogue score.

**Table 4:** Association between characteristics of patients with quality of life (EQ-5D Score).

Variable	EQ-5D Score		*p*-value in bivariate analysis	*p*-value in multivariate analysis	Unadjusted OR (95% CI)	Adjusted OR (95% CI)
	Median	IQR				
Sex						
Female	0.79	0.27	0.773	-	0.60 (0.14–2.64)	-
Male	0.85	0.36				
Age						
< 44 years	0.79	0.29	0.627	-	0.87 (0.41–1.84)	-
≥ 44 years	0.80	0.23				
Ethnicity						
Sundanese	0.79	0.27	0.186^b^	0.750	0.79 (0.34–1.85)	0.85 (0.32–2.28)
Non-Sundanese	0.81	0.26				
Smoking history						
Non-smoker	0.79	0.27	0.496	-	0.39 (0.07–2.12)	-
Smoker	0.91	0.17				
Alcoholic history						
Non-alcoholic	0.79	0.27	0.883	-	0.51 (0.04–5.79)	-
Former alcoholic	0.87	0				
Age of onset						
≤55	0.80	0.27	0.782	-	1.41 (0.30–6.63)	
>55	0.79	0.38				
Chronic comorbidities						
No	0.83	0.19	0.001*	0.005*	3.31 (1.151–7.23) *	3.65 (1.50–8.93) *
Yes	0.75	0.33				
Disease duration						
< 3 years	0.79	0.30	0.464	-	0.87 (0.41–1.84)	-
≥ 3 years	0.79	0.21				
Current therapy						
MTX monotherapy	0.80	0.24	0.829	-	1.12 (0.50–2.51)	-
Non-MTX monotherapy	0.79	0.28			1.41 (00.28–7.18)	
2 or 3 DMARDs	0.78	0.33			Ref.	
Involuntary discontinuation of MTX						
No discontinuation history	0.79	0.27	0.950	-	0.78 (0.33–1.87)	-
Had discontinuation history	0.80	0.28				
Rheumatoid factor						
Reactive	0.79	0.32	0.306	-	0.87 (0.41–1.85)	-
Non-reactive /Unknown	0.80	0.21				
Duration of MTX therapy						
< 6 months	0.78	0.28	0.773	-	0.78 (0.32–1.88)	-
≥ 6 months	0.80	0.26				
Current MTX weekly dose						
< 5 tablets/week	0.78	0.28	0.737	-	0.75 (0.35–1.59)	-
≥ 5 tablets/week	0.82	0.24				
Methylprednisolone daily dose						
<4 mg	0.84	0.28	0.139 ^b^	0.932	1.68 (0.76–3.75)	1.04 (0.41–2.63)
≥ 4 mg	0.79	0.26				
Disease activity						
Lower disease activity group	0.83	0.17	<0.001*	<0.001*	6.03 (2.32–15.69) *	6.33 (2.29–17.51) *
Higher disease activity group	0.63	0.25				

Higher disease activity group = patients with moderate disease activity (MDA) and high disease activity (HDA); Bivariate analysis was conducted using Mann–Whitney U test (for 2 independent groups) or Kruskal–Wallis test (for 3 independent groups); Multivariate analysis and odds ratio calculation were conducted using binary logistic regression; *p < 0.05 considered statistically significant; bold values indicate statistically significant results; ^b^ variable with *p* < 0.25 in bivariate analysis was included into multivariate model.

SD, standard deviation; IQR, interquartile range; MTX, methotrexate; DMARDs, disease-modifying antirheumatic drugs; OR, odds ratio.

Additionally, multivariate logistic regression demonstrated that RA patients with no chronic comorbidity were 3.65 times more likely to have higher HRQoL (OR = 3.65; 95% CI 1.50–8.93). Likewise, in the lower disease activity group, which was 6.33 times more likely to have higher HRQoL (OR = 6.33; CI 95% 2.29–17.51) (Table 4).

## 4 Discussion

This study illustrates the association between RA and reduced quality of life in the Indonesian setting, which was attributable to disease severity and the presence of comorbidity. It also highlights that pain or discomfort—regardless of severity—was the domain most significantly affecting patients with RA, with statistically significant differences observed across disease activity groups. This finding aligns with an observational study conducted in Thailand, which reported that 70.5% of patients experienced slight to extreme pain or discomfort (13). Another cross-sectional study with the EQ-5D questionnaire among Pakistani RA patients also demonstrated that most patients (95.7%) reported pain or physical problems (14).

Previous studies have also shown that pain in RA significantly impacts various aspects of life, including mood, mobility, work performance, relationships, sleep quality, and overall life satisfaction (15). In the early stages of the disease, patients may experience minimal pain and cartilage damage. However, as RA progresses, inflammation and joint deformities often develop, leading to persistent pain (16). A prior analysis of six studies demonstrated a strong, dynamic relationship between RA disease activity and pain levels. Notably, RA-related pain intensity can fluctuate rapidly over time (17). Our findings are consistent with these previous reports.

Additionally, this study found no significant differences in the anxiety/depression domain across disease activity groups (*p* = 0.391). This result is in line with a previous study by Katchmart, which reported a similar prevalence of anxiety across different RA disease states (13). A bidirectional relationship between depression and RA may explain this finding: individuals with RA are more likely to experience depression, while those with depression have a higher risk of developing RA. A longitudinal study showed that RA patients had a 1.69 times higher rate of depression, and depressed individuals had a 1.65 times higher rate of RA incidence (18). This connection may be mediated by systemic inflammation involving pro-inflammatory cytokines (19). Therefore, a patient’s well-being is influenced not only by physical pain but also by psychological factors such as anxiety and depression, regardless of disease severity.

This study also confirmed that the health-related quality of life (HRQoL) of RA patients is lower than that of the general population in Indonesia (10). Moreover, their HRQoL scores were similar to or lower than those of patients with type 2 diabetes mellitus, one of the most prevalent chronic diseases in the country. Reported scores in diabetic populations include 0.86 (95% CI, 0.83–0.88; n = 206), 0.77 (95% CI, 0.75–0.79, *n* = 907) (20, 21), and 0.75 ± 0.22 (*n* = 86) (22). A meta-analysis evaluating HRQoL in RA patients across Asia found even lower pooled EQ-5D index and EQ-5D VAS scores: 0.66 (95% CI, 0.63–0.69) and 61.21 (95% CI, 50.73–71.69), respectively, with high heterogeneity (I^2^ = 99.65 and 99.56%) (23). These cross-disease and cross-regional comparisons underscore the substantial impact of RA on patient well-being.

Furthermore, our findings support the association between higher disease activity and lower HRQoL (*p* < 0.001). This aligns with a previous meta-analysis, which found that EQ-5D scores declined as disease activity increased: the pooled scores were 0.78 (95% CI: 0.65–0.90) in remission, 0.73 (95% CI: 0.65–0.80) in low disease activity (LDA), 0.53 (95% CI: 0.32–0.74) in moderate disease activity (MDA), and 0.47 (95% CI: 0.32–0.62) in high disease activity (HDA) (23). Similarly, a meta-analysis by Matcham et al. using the SF-36 instrument concluded that RA has a considerable impact on HRQoL, with pooled mean scores of 34.1 (95% CI: 22.0–46.1) for the physical component summary and 45.6 (95% CI: 30.3–60.8) for the mental component summary (24). Thus, RA disease activity demonstrably reduces HRQoL as assessed by both EQ-5D and SF-36 instruments.

Disease activity reflected the effectiveness of treatment. In this study, majority of patients achieved low disease activity or remission while receiving methotrexate, with or without other csDMARDs. The median dose was 12.5 mg/week, consistent with the standard MTX dose for RA treatment in Indonesia and internationally (7.5–25 mg/week) (25). It was suggested that in general, Asian RA patients showed higher responsiveness to MTX than Caucasians, which was partly predisposed by genetic factor (26). Ideally, when csDMARDs are inadequate, bDMARDs should be added (25, 27). However, in this study, no subjects received biological DMARDs as routine treatment due to reimbursement restrictions.

This study also found that chronic comorbidity, such as cardiovascular and metabolic diseases, was a clinical characteristic associated with HRQoL of RA patients. This finding supported the existing systematic review demonstrating worse HRQoL among patients with comorbidities, especially cardiovascular disease, hypertension, and diabetes (28). Additionally, clinical factors not assessed in this study—such as history of coronary artery disease, functional impairment, anxiety, and depression—have also been identified as determinants of EQ-5D scores (13, 29). The finding highlights the need of managing comorbidities along with RA treatment.

Earlier studies had identified an association between demographic characteristics and HRQoL. A meta-analysis by Haridoss et al. reported lower EQ-5D scores among female patients (23). Involvement of genetic factors and hormones may explain the greater burden in women (30). However, in this research, uneven sex distribution limits the generalizability of sex-related findings. Moreover, the meta-analysis also reported that older age had a greater likelihood of lower HRQoL, which was not observed in this study (30). Our findings suggest potential contextual differences, particularly related to socio-demographics. Financial-related burden has been widely discussed as one of contextual factor of HRQoL in Indonesia (31, 32).

This study confirms the relationship between HRQoL and RA disease activity in Indonesian patients, which had been demonstrated in previous studies worldwide. This study has several limitations. Firstly, data collection was restricted to Bandung, one of Indonesia’s most populous cities, but this single-center approach may limit the generalizability of findings to the broader Indonesian RA population. Secondly, an imbalanced proportion of male and female patients prevents reliable conclusion of sex-related findings. Therefore, it should be interpreted carefully. Additionally, mental health and functional disability were not evaluated in this study.

Finally, this study represents real-world data of RA patients in Indonesian setting. Future studies with larger, more diverse samples, and longitudinal data collection are needed to provide a more comprehensive perspective. This study also highlights the importance of incorporating utility scores as complementary endpoints in RA monitoring and treatment evaluation to better capture the overall well-being of patients. Moreover, HRQoL assessments can contribute valuable insights for future cost-effectiveness analyses.

## 5 Conclusion

In conclusion, this cross-sectional study suggests that higher disease activity level and the presence of chronic comorbidity were negatively associated with HRQoL among RA patients in Bandung, Indonesia. Disease progression not only leads to physical pain but also significantly disrupts multiple dimensions of patients’ quality of life. Therefore, assessing HRQoL should be an integral part of RA treatment, along with management of comorbidities. A more comprehensive study is warranted to obtain broader insight into RA patients in Indonesia.

## Data Availability

The datasets presented in this article are not readily available because they can only be accessed from the authors upon reasonable request. Requests to access the datasets should be directed to Yudisia Ausi, yudisia13001@mail.unpad.ac.id.
